# Chronic bacterial inflammation induces prostatic intraepithelial neoplasia in mouse prostate

**DOI:** 10.1038/sj.bjc.6605370

**Published:** 2009-10-20

**Authors:** J E Elkahwaji, R J Hauke, C M Brawner

**Affiliations:** 1 Department of Internal Medicine, Section of Adult Oncology and Hematology and GU Oncology Research Laboratory, University of Nebraska Medical Center, Omaha, NE, USA

**Keywords:** chronic bacterial inflammation, *π* class glutathione-*S*-transferase, prostatic intraepithelial neoplasia, phosphatase and tensin homologue, oxidative stress, prostate cancer

## Abstract

**Background:**

Although the aetiology of prostate cancer remains unknown, we hypothesised that chronic bacterial insult has a major role in prostate carcinogenesis.

**Methods:**

Male C3H/HeOuJ mice, infected with phosphate-buffered saline or *Escherichia coli* bacteria, were killed at 5 days, or at 12 or 26 weeks. Harvested prostate tissues were evaluated for inflammatory responses and immunostained for neoplastic transformation markers.

**Results:**

All infected mice developed bacterial prostatitis. Control mice had no prostate infections or inflammation. Mice infected for 5 days showed foci of acute inflammation with infiltrating neutrophils and epithelial necrotic debris in the prostatic glandular lumen. All mice infected for 12 weeks had evidence of chronic inflammation with dense inflammatory infiltrates in the stroma. The prostatic epithelium showed varying degrees of atypical hyperplasia with increased epithelial cell layers and cytological atypia. At 26 weeks, the dysplastic changes were more pronounced and mimicked a prostatic intraepithelial neoplasia and high-grade dysplasia. Prostatic glands exhibiting reactive dysplasia had a stronger staining for oxidative DNA damage, increased epithelial cell proliferation, and a decrease in androgen receptor, *GSTP1*, *p27*^*Kip1*^, and *PTEN* expression, when compared with control prostate glands.

**Conclusion:**

These data demonstrate that chronic inflammation induces focal prostatic glandular atypia and suggest a potential linkage between inflammation and prostatic neoplasia.

In spite of progress in diagnosis and treatment, prostate cancer (CaP) remains a major public health problem in the male population and the second leading cause of death among men in the United States and in many Western industrialised countries. Prostate cancer is predominantly a disease in men over 40 years of age, and its incidence increases steeply in the seventh decade of life. In 2008, an estimated 186 360 men were diagnosed with CaP, and it was estimated that 28 660 deaths because of CaP occured (Jemal *et al*, 2008). Widespread screenings for prostate-specific antigen, digital rectal examination, and needle biopsy, as well as standard treatment already in clinical use, have enhanced patient's survival by improving the detection of early and localised disease (Hankey *et al*, 1999; Bartsch *et al*, 2008). In addition, it is likely that better preventive measures will reduce the incidence of CaP (Djavan *et al*, 2004). In contrast, there is still no effective cure for those patients having extra-prostatic invasion, distant metastasis, or hormone refractory CaP.

Although the exact aetiology of CaP is largely unknown, this seems to be a multifactorial disease in which several environmental and genetic factors are likely to be involved (Lichtenstein *et al*, 2000; Migliore and Coppede, 2002). Epidemiological studies have shown that the incidence of CaP shows age, race, diet, and geographical dependence (Lichtenstein *et al*, 2000; Nelson *et al*, 2002), with North Americans and Western Europeans showing high rates of disease, whereas men in Asian nations experience lower rates (Hsing *et al*, 2000). In addition to disparities in rates between different populations and ethnic groups, three susceptible genes, ribonuclease L (*RNaseL*) (Rokman *et al*, 2002), macrophage scavenger receptor 1 (*MSR1*) (Xu *et al*, 2002), and toll-like receptor-4 (*TLR-4*) (Sun *et al*, 2007), have been associated with familial CaP and all functions in host immune responses or in host protection against cellular and genomic damage mediated by the infection itself or by the inflammatory oxidants secreted by leukocyte-infiltrating cells in response to infection. Other aetiological factors such as high androgen levels (Feldman and Feldman, 2001), growth factor overexpression (Pollak, 2008), dietary carcinogens (Cohen *et al*, 2000; Freeman *et al*, 2000; Kolonel *et al*, 2000; Kooiman *et al*, 2000; Chan and Giovannucci, 2001; Kolonel, 2001; Shapiro *et al*, 2001), sexually transmitted diseases (Strickler and Goedert, 2001; Sutcliffe and Platz, 2008), and other infectious agents (Dennis *et al*, 2002) may be associated with increased CaP risk. Moreover, somatic targets of genomic damage and inherited polymorphic variants of genes mediating androgen action may enhance the progression of CaP (Nelson *et al*, 2003).

In the last decade, advanced cancer research has pointed out several cancer-causing factors, including spontaneous replication errors in DNA, endogenous and exogenous carcinogenic substances, irradiation such as ultraviolet and transduction of viral oncogenes, and infection/inflammation. The role of infection/inflammation in the initiation and progression of cancer has been an area of intense scientific interest and usually considered from the perspective that persistent inflammation in the context of chronic infection or tissue injury might promote cell transformation through DNA damage or that tumour cells produce proinflammatory factors that derive chronic inflammation and tumour growth. It is estimated that 20–25% of all human cancers are caused by chronic infection and inflammation (De Marzo *et al*, 2007).

In the set of CaP, chronic prostatic inflammation is increasingly often discussed as a critical component of tumour carcinogenesis by generating a pathologically conducive microenvironment that may favour the initiation and progression of cancer. A sustained inflammatory microenvironment provides a constant supply of various reactive nitrogen and oxygen species, reactive aldehydes, cytokines, chemokines, and growth factors, which can alter crucial biological processes responsible for maintaining normal cellular homeostasis, leading to genomic instability and risk of CaP development (Balkwill and Mantovani, 2001; Coussens and Werb, 2002; Gupta and Dubois, 2002; Philip *et al*, 2004; Hussain and Harris, 2007). Moreover, proliferative inflammatory atrophy (PIA), areas of epithelial proliferation associated with chronic inflammation, has been found to be closely associated with areas of prostatic intraepithelial neoplasia (PIN) and CaP (De Marzo *et al*, 1999b). Multiple epidemiological and clinical studies have identified an association between specific bacterial infections and CaP (Dennis *et al*, 2002). Nevertheless, a recent meta-analysis found a small increased risk in the relative risk of CaP in men with a previous medical history of clinical or symptomatic prostatitis (Dennis and Dawson, 2002). Other investigations of infectious agents in the aetiology of chronic prostatitis/chronic pelvic pain syndrome have shown a correlation between inflammation and bacterial genes detected in prostate biopsy specimens, indicating that localised prostatic inflammation induced by chronic bacterial colonisation may be attributed to CaP and could have an important role in aetiology (Hochreiter *et al*, 2002). We postulated that chronic bacterial insult is an important source of prostatic inflammation and has a role in pre-neoplastic events. This hypothesis was tested in a mouse model of chronic bacterial prostatitis, in which biomarkers and histopathological changes associated with cellular oxidative stress and neoplastic transformation were evaluated.

## Materials and methods

### Animals

Male C3H/HeOuJ mice, 10–12 weeks old, were purchased from Jackson laboratories (Bar Harbor, Maine, NY, USA) and housed in accordance with the guidelines of the Association for the Assessment and Accreditation of Laboratory Animal Care. The animals were maintained under normal conditions at ambient temperature and relative humidity and fed with standard diet provided by Jackson Laboratories. Tap water was provided *ad libitum*. All experimental procedures were approved by the University of Nebraska Medical Center Animal Care and Use Committee Guidelines.

### *Escherichia coli* strain and bacterial infection

*Escherichia coli* bacteria were grown from frozen stock by two passages in tryptose broth (Difco Laboratories, Detroit, MI, USA), washed with sterile phosphate-buffered saline (PBS) by centrifugation, and resuspended to a concentration of 1 × 10^8^ bacteria ml^−1^. A group of six mice were deprived of water for 1 h and had urine expressed from their bladders immediately before inoculation. The mice were anaesthetised with isoflurane and inoculated intraurethrally, using a lubricated sterile polyethylene catheter (Intramedic PE-10) (BD, Diagnostic System, Sparks, MD, USA), with 20 *μ*l of bacterial inoculum resulting in a dose of 2 × 10^6^ *E. coli* per mouse. Control mice were inoculated with an equal volume of PBS. The animals were allowed to recover from anaesthesia and were given free access to water 1 h later. The mice were killed at 5 days, or 12 or 26 weeks after inoculation. The prostate of each animal was removed intact and bisected in the mid-sagittal plane. One half was used for bacterial analysis as described previously (Elkahwaji *et al*, 2005). The remaining half was fixed in 10% formalin, and evaluated by light microscopy after haematoxylin and eosin, immunohistochemistry, or immunofluorescence staining.

### Haematoxylin and eosin staining

All prostate tissues obtained from control and *E. coli*-infected animals were processed routinely through graded concentrations of alcohol and embedded in paraffin blocks. Five-micron sections were cut, deparaffinised, and rehydrated in increasing concentrations of ethanol. Tissues were rinsed with deionised water for 15 s and stained with haematoxylin for 4 min. Samples were dehydrated with 70% ethanol; followed by 95% ethanol for less than 5 s. Eosin was applied for 2 min. After staining, samples were dehydrated in an increasing series of ethanol (95% ethanol for 5 s, 100% ethanol for 5 s, and then 100% ethanol for 5 s) and cleared in xylene (twice for less than 1 min each and once for 2 min).

### *In vivo* 5-bromo-2-deoxy-uridine Labelling and immunostaining

Male C3H/HeOuJ mice were treated by IP injection with 0.2 ml of 5-bromo-2-deoxy-uridine (BrdU) (10 mM) per mouse as instructed (Roche Diagnostic GmbH, Mannheim, Germany). The mice were killed 1 h later and the prostates of both infected and control groups were placed into a separate section of a multi-chamber cassette for tissue processing, embedding, and sectioning. After deparaffinisation and antigen retrieval, the prostatic cells were immunostained using the BrdU Labeling and Detection Kit II (Roche Diagnostic GmbH). Goat anti-mouse-Alexa 546-conjugated antibody (Molecular Probes, Carlsbad, CA, USA) was used to visualise the BrdU-stained prostatic cells. Sections were mounted with Vectashield Hardset mounting media+DAPI counterstain (Vector Laboratories Inc., Burlingame, CA, USA), and images were taken using an Olympus model BX51 fluorescent microscope equipped with DAPI and rhodamine filters and Spot Advanced software v. 3.5.2 (Hitschfel Instruments, Inc., St Louis, MO, USA).

### Immunofluorescence staining for oxidative DNA damage

Staining for oxidative DNA damage was carried out by an immunofluorescence technique using a primary monoclonal antibody against 8-hydroxy-deoxyguanosine (8-OH-dG) and by applying the method of Yarborough *et al* (1996) with some modifications. Briefly, after deparaffinisation and rehydratation, prostate sections were treated sequentially with Ribonuclease A (RNAse) (Sigma Chemical Co., St Louis, MO, USA) and Proteinase K solution (Chemicon International, Temecula, CA, USA) for 1 h and 30 min, respectively, at room temperature, followed by two PBS washes for 1 min each. After RNAse and Proteinase K treatment, the sections were sequentially treated with HCl solution for 7 min and with 50 mM Tris base solution to neutralise the specimens and were then submerged in BioGenex Super Sensitive Wash Buffer (Biogenex, San Ramon, CA, USA) at room temperature. Prostate sections were incubated in Zymed Goat Serum (10%) (Zymed Laboratories, Inc., San Francisco, CA, USA) for 20 min at room temperature to block nonspecific binding, then incubated overnight at 4°C with monoclonal anti-8-OH-dG mouse antibody (1 : 100, QED) or with mouse immunoglobulin (Ig) G1 clone P3 isotype control (1 : 7500, eBioscience, San Diego, CA, USA). After the overnight incubation, a secondary antibody was applied (1 : 100, Goat anti-mouse IgG labelled with Alex Fluor 488 (Molecular Probes Inc., Eugene, OR, USA)) for 1 h at room temperature. Slides were washed twice with BioGenex Super Sensitive Wash Buffer, tapped dry, mounted with Prolong Gold Antifade Reagent (Molecular Probes Inc.), coverslipped, then sealed with a clear lacquer sealant. Images were captured using a fully automated Leica DMXRA2 microscope (North Central Instruments, Plymouth, MN, USA).

### Immunofluorescence staining in paraffin section for *π*-class glutathione-*S*-transferase, cyclin-dependent kinase inhibitor (*p27*^*Kip1*^), and tumour suppressor gene phosphatase and tensin homologue

Five-micron prostate sections were cut, deparaffinised in xylene, and rehydrated in a graded concentration of alcohol and distilled water. Antigen retrieval was performed in citrate buffer (pH=6) using an electric pressure cooker (Biocare Medical, Walnut Creek, CA, USA) at 120°C for 2 min (Norton *et al*, 1994), followed by rehydration in PBS and incubation in Zymed Goat Serum (10%) for 20 min at room temperature to block nonspecific binding. Different prostate sections were incubated overnight at 4°C with monoclonal anti-*p27*^*Kip1*^ mouse antibody (1 : 20, Vector Laboratories Inc.), polyclonal anti-GSTP1 rabbit antibody (1 : 100, Vector Laboratories Inc.), or polyclonal anti-PTEN rabbit antibody (1 : 200, Abcam Inc. Cambridge, MA, USA). Control prostate sections were incubated with rabbit or mouse isotype control (Invitrogen Corporation, Camarillo, CA, USA). After the overnight incubation, a secondary antibody was applied (1 : 200, Goat anti-mouse IgG labelled with Alex Fluor 488 or Goat anti-rabbit IgG labelled with Alex Fluor 568 (Molecular Probes Inc.)) for 1 h at room temperature. Slides were washed twice with BioGenex Super Sensitive Wash Buffer, tapped dry, mounted with Prolong Gold Antifade Reagent (Molecular Probes Inc.), coverslipped, then sealed with a clear lacquer sealant. Images were captured using a fully automated Leica DMXRA2 microscope (North Central Instruments).

### Immunohistochemistry in paraffin section for androgen receptor

Five-micron sections were cut, deparaffinised in xylene, and rehydrated in a graded concentration of alcohol and distilled water. Antigen retrieval was performed in citrate buffer (pH=6) using an electric pressure cooker (Biocare Medical) at 120°C for 2 min (Norton *et al*, 1994), and endogenous peroxidase activity was blocked with 3% hydrogen peroxide in methanol for 30 min, followed by rehydration in PBS and incubation with 5% normal goat serum for 30 min at room temperature to bind nonspecific antigens. Prostate tissue sections were then incubated for 1 h with rabbit polyclonal antibody against androgen receptor (AR, 1/20; Novocastra, Burlingame, CA, USA). After washing unbound primary antibody, sections were treated for 45 min at room temperature with specific commercial biotinylated secondary anti-IgG, followed by avidin coupled to biotinylated horseradish peroxidase, according to the manufacturer's instructions (the LSAB2 kit for rabbit primary antibodies, DAKO Corporation, Carpinteria, CA, USA). Diaminobenzidine was applied as the chromogenic peroxidase substrate for 3–5 min at room temperature to reveal staining. Sections were counterstained with haematoxylin for 1–2 min and analysed by standard light microscopy. Specificity was verified by negative control reactions in which the primary antibody was replaced with normal rabbit serum.

## Results

In this study, we assessed the prostate infection intensities at 5 days, and at 12 and 26 weeks after infection (Table 1) in C3H/HeOuJ male mice after inoculation with PBS (*n*=18) or with 2 × 10^6^ uropathogenic *E. coli* bacteria (*n*=36). The quantitative colony-forming unit (CFU) per mg of prostate tissue showed no significant prostate infections in PBS control mice at either time points. In contrast, all infected C3H/HeOuJ mice with 2 × 10^6^ bacteria developed acute bacterial prostatitis at 5 days, and active chronic bacterial prostatitis at both 12 and 26 weeks. The intensity of prostate infection was more pronounced at 26 weeks when compared with the infection intensity found at 5 days and 12 weeks (138, 9375, and 30 785 CFU per mg of prostate tissue, at 5 days, and at 12 and 26 weeks, respectively).

**Figure 1:**
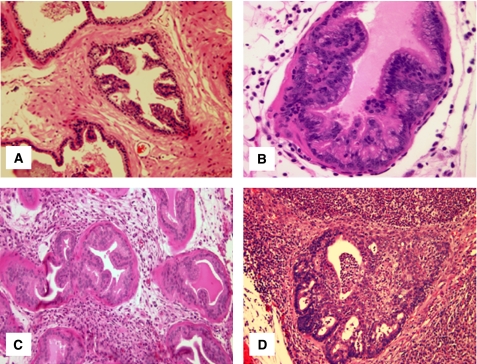
Acute and chronic prostatic inflammation induced by *E. coli* bacterial infection in C3H/HeOuJ mice. (**A**) PBS-control prostate. (**B**) Acute prostatic inflammation 5 days after infection characterised by acute inflammatory cells. (**C**) Mild-to-moderate chronic inflammation at 12 weeks after infection associated with variant degrees of atypical hyperplasia and dysplasia. (**D**) Severe chronic inflammation at 26 weeks associated with severe dysplasia and cytological atypia (magnification × 40).

**Figure 2:**
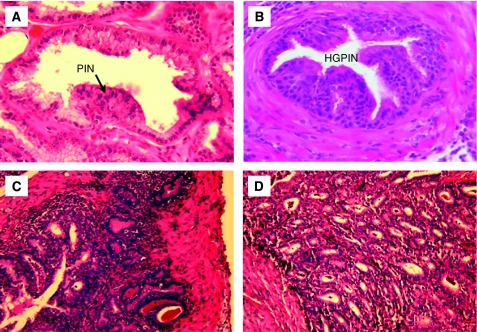
Chronic bacterial inflammation induces dysplastic changes in mouse prostate. (**A** and **B**) Chronic inflammation induces high-grade prostatic intraepithelial neoplasia at 12 weeks after inoculation. (**C** and **D**) Dysplastic changes associated with severe chronic inflammation observed at 26 weeks after infection and mimicking a high-grade dysplasia and an invasive carcinoma (magnification × 40).

In addition, our bacteriological studies were supplemented by histological examination of prostate tissues collected at 5 days, and at 12 and 26 weeks after instillation of PBS or 2 × 10^6^ uropathogenic *E. coli* bacteria. Control mice inoculated with PBS revealed a normal prostate architecture, normal epithelial morphology, and absence of inflammatory cell infiltrate (Figure 1A). In contrast, prostate tissues obtained from C3H/HeOuJ mice inoculated with uropathogenic bacteria for 5 days exhibited multiple foci of acute inflammation associated with acute inflammatory cells (neutrophils), epithelial necrotic debris, and an abundant shedding of epithelial cells in the prostatic glandular lumen (Figure 1B). At 12 weeks after infection, acute inflammation decreased and focal residual acute inflammation was present in prostatic glands. In addition, focal mild-to-moderate chronic inflammation was present in the stroma. Most significantly, the prostatic glandular epithelium showed varying degrees of atypical hyperplasia with increased epithelial layers, severe cytological atypia, and dysplastic changes of glandular architecture (Figure 1C). The prostatic glands were distorted and became irregular with a bulging profile associated with prominent chronic inflammation, whereas acute inflammation subsided. Sites of intense inflammation were associated with moderate or dense lymphocytic infiltrate in the periglandular stroma and shedding of epithelial cells into the glandular lumen (Figure 1D). The dysplastic changes observed at this stage mimic a high-grade PIN, a potential precursor to CaP in human prostate (Figure 2A and B). Furthermore, prostate tissues obtained from additional mice and examined histologically 26 weeks after inoculation showed intense chronic inflammatory changes similar to those evident at 12 weeks after inoculation, but the dysplastic changes were more pronounced at this stage, nearly replacing the normal glandular architecture and mimicking a high-grade dysplasia or a cancer-like lesion (carcinoma *in situ*/invasive carcinoma) (Figure 2C and D). These data suggest that chronic bacterial infection induced a PIN lesion associated with severe chronic inflammation and this prostate lesion is similar to the PIN lesion described by De Marzo and colleagues that occurs in close proximity to foci of chronic inflammation and that constitute a potential precursor to human prostate adenocarcinoma.

**Figure 3:**
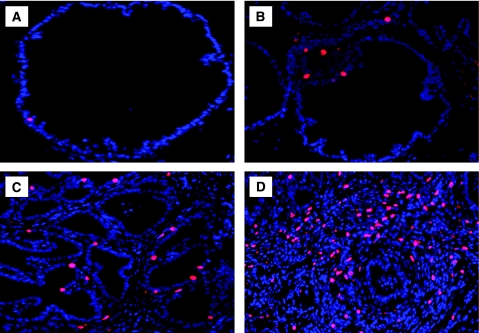
Prostatic epithelial cell proliferation induced by chronic inflammation. (**A**) PBS-control tissues show sparse staining for BrdU. (**B** and **C**) Prostate tissues exhibiting a varying degree of atypical hyperplasia and dysplasia at 12 weeks after inoculation show numerous BrdU-stained epithelial nuclei. (**D**) Prostate tissue exhibiting high-grade dysplasia or cancer-like lesion at 26 weeks after infection show an increased number of BrdU-stained epithelial nuclei as compared with PBS-control and 12 week-infected prostate tissues (magnification × 40).

**Figure 4:**
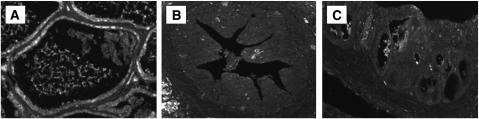
Expression of the cyclin-dependent kinase inhibitor (*p27*^*Kip1*^) in mouse prostate. (**A**) PBS-control prostate showing a positive staining for *p27*^*Kip1*^, a suppressor of prostatic epithelial cell proliferation. (**B** and **C**) Prostate tissues exhibiting severe dysplastic changes and mimicking a high-grade PIN and carcinoma *in situ*, showing a negative staining for *p27*^*Kip1*^ when compared with PBS-control prostate (magnification × 20).

To determine whether the PIN lesion and severe dysplatic changes associated with chronic inflammation were because of an increase in prostatic epithelial cell proliferation, mice were treated with BrdU 1 h before being killed, then a quantitative analysis of BrdU incorporated into the newly synthesised DNA of replicating prostatic epithelial cells was performed (Figure 3). We observed a very low labelling index in PBS prostate tissue. Staining for BrdU showed a clear increase in prostatic epithelial cell proliferation in glands exhibiting PIN lesion and severe dysplasia observed at 12 and 26 weeks after infection. Moreover, the prostate tissues exhibiting PIN lesions and high severe dysplasia showed a clear decrease in *p27*^*Kip1*^ expression, a suppressor of prostatic epithelial cell proliferation, when compared with PBS-control prostate (Figure 4). These results support the conclusion that PIN lesion and the dysplastic changes associated with chronic bacterial infection and inflammation occurred in conjunction with increased prostatic epithelial cell proliferation.

**Figure 5:**
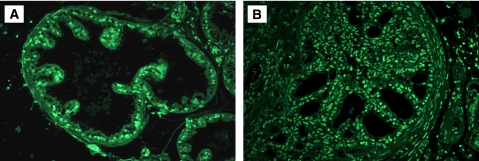
Chronic bacterial inflammation induces oxidative DNA damage in mouse prostate. (**A**) Prostate tissues after PBS treatment showing a low nuclear staining for 8-hydroxy-2′-deoxyguanosine (8-OH-dG), a marker for oxidative DNA damage. (**B**) Prostate tissues exhibiting a varying degree of atypical hyperplasia and severe dysplasia after bacterial infection showing an increased staining for 8-OH-dG in prostatic epithelial nuclei (magnification × 40).

**Figure 6:**
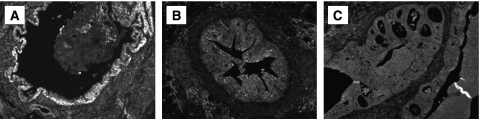
Expression of the *π*-class glutathione-*S*-transferase (*GSTP1*) in mouse prostate. (**A**) PBS-control prostate showing a high level of *GSTP1,* the function of which induces phase II detoxifying enzymes for reactive oxygen species and organic electrophiles. (**B** and **C**) Prostate tissues exhibiting a high-grade PIN and carcinoma *in situ* showing an absence of *GSTP1* expression when compared with PBS-control prostate (magnification × 20).

The association between CaP risk and oxidative stress has been recognised, and epidemiological, experimental, and clinical studies have unequivocally proven a role of oxidative stress (reactive oxygen and nitrogen species) in the development and progression of the disease. These reactive oxygen species (ROS), in the absence of *GSTP1*, which function as an inducible phase II detoxifying enzyme for ROS and organic electrophiles, induce tissue injury and DNA damage, which are general manifestations of pathological conditions associated with infection, inflammation, and epithelial cell proliferation. In this study, we performed an immunofluorescence staining for the oxidative stress-associated marker 8-OH-dG (Figure 5) and for the caretaker gene *GSTP1* to ensure that PIN lesion and the dysplastic changes associated with chronic bacterial inflammation occurred in a microenvironment rich in ROS. Nuclear staining for 8-OH-dG was rare in PBS control prostate. However, prostate tissues exhibiting PIN lesion and severe dysplasia demonstrated an increased nuclear staining for oxidative DNA damage (Figure 5) and an absence of *GSTP1* expression (Figure 6) when compared with the PBS-control prostate. These data suggest that PIN lesion and severe dysplasia mimicking a cancer-like lesion arise as a consequence of the regenerative proliferation of prostate epithelial cells in response to injury caused by inflammatory oxidants in response to bacterial infection.

**Figure 7:**
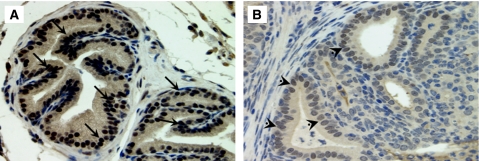
Immunstaining for the androgen receptor (AR) in mouse prostate. (**A**) PBS-contol prostate showing a positive staining for androgen receptor (arrows). (**B**) Prostate tissues exhibiting severe dysplasia after bacterial infection showing a decreased staining for androgen receptor as compared with PBS-control prostate (arrowhead) (magnification × 40).

**Figure 8:**
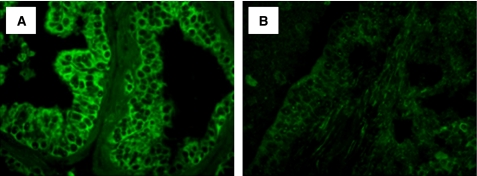
Expression of the tumour suppressor gene phosphatase and tensin homologue (PTEN) in mouse prostate. (**A**) PBS-control prostate showing a high level of PTEN expression. (**B**) Prostate tissue exhibiting severe dysplastic changes and mimicking high-grade PIN and carcinoma *in situ* showing a loss of expression of PTEN when compared with PBS-control prostate (magnification × 40).

The progression from PIN lesion to localised then advanced CaP occurred in an androgen-independent manner. This phenomenon reflects the ability of prostatic epithelial cells to sustain growth and proliferation even in the absence of androgen, thus acquiring androgen independency and an aggressive phenotype. Similarly, this progression occurs during some genomic alterations resulting in loss or inactivation of many tumour suppressor genes such as PTEN, a phosphatase and tensin homologue involved in several signalling pathways that regulate cell growth and survival. In this study, we performed an immunostaining for AR and PTEN expression to determine whether the dysplastic changes mimicking a cancer-like lesion acquire an androgen independency (Figure 7) and to ensure that lost or inactivated tumour suppressor gene PTEN will promote the progression of prostate carcinogenesis (Figure 8). A decrease in AR and a loss of PTEN expressions were observed in the atypical prostate gland as compared with normal and simple hyperplastic glands, suggesting that the papillary form of the neoplastic lesion induced by chronic bacterial inflammation may progress towards an invasive cancer by bypassing androgens and the AR, and indeed occurs during PTEN loss or inactivation.

## Discussion

Prostate cancer remains a major concern of public health in the United States. Tremendous morbidity, mortality, and economic costs are associated with screening, treatment, and palliation of the disease. Aetiological factors that initiate and enhance the progression of this malignancy are beginning to emerge, with strong evidence that chronic inflammation and uncontrolled cell proliferation within the prostate are linked to initiation and neoplastic conversion. This evidence suggests that dietary strategies targeting inflammation and proliferation would be effective in limiting CaP development.

Histopathological studies of human prostate reveal a prevalence of inflammatory changes in the peripheral zone of the prostate, the most common site of tumourigenesis (McNeal, 1988). In addition, molecular epidemiological studies indicate a high prevalence of colonisation of the prostate, with inconclusively identified bacterial species in men with chronic prostatitis (Hochreiter *et al*, 2002). Because *E. coli* is the most common pathogen identified in acute prostatitis in humans (Mitsumori *et al*, 1999) and is able to induce acute and chronic bacterial prostatitis in rats (Nickel *et al*, 1990; Rippere-Lampe *et al*, 2001), we developed a new mouse model of bacterial prostatitis to study the relationships between bacterial infection and inflammation, oxidative stress, and prostatic carcinogenesis. In this study, all C3H/HeOuJ inbred male mice, known to be highly susceptible to infection, developed acute bacterial prostatitis at 5 days and severe, chronic bacterial prostatitis at 12 and 26 weeks after inoculation with *E. coli* bacteria. In addition, *E. coli* infection induced a focal acute inflammation within the prostate at day 5 and chronic prostatic inflammation at 12 and 26 weeks after infection. Most significantly, the prostatic glandular epithelium showed atypical hyperplasia associated with increased epithelial cell layers, cytological atypia, and dysplastic changes of glandular architecture. In contrast, PBS control mice had no prostate infection and inflammation at any time point. These results are consistent with those observed in rats (Reznik *et al*, 1981; Wilson *et al*, 1990), in which prostatic inflammation, accompanied by focal epithelial atrophy, may also contribute to the development of CaP and reinforced the potential linkage between prostatic inflammation and aetiology of CaP.

Recently, De Marzo *et al* (1999b) proposed a hypothesis of CaP development on the basis of chronic inflammation, ROS-induced genomic damage, and neoplastic transformation. The central concept of this hypothesis is that carcinogenesis results from repeated tissue damage and regeneration in the presence of ROS and reactive nitrogen species (RNS) released from inflammatory cells. In both acute and chronic infections, infiltrating leukocytes and phagocytic cells destroy bacteria, parasites, and virus-infected cells by producing ROS and RNS (Ames *et al*, 1995). The presence of ROS and RNS causes oxidative damage to cellular DNA, proteins, and lipids, leading to genetic mutations, chronic cell killing, and compensatory cell division. It is conceivable that repeated host inflammatory responses due to long-term bacterial colonisation could provide one source of ROS-producing neutrophils. Reactive molecules would be expected to interact with genomic or mitochondrial DNA in normal prostate epithelial cells, causing genomic alterations such as point mutations, deletions, or rearrangements that accumulate and become permanent in the genome of proliferating cells (Oberley, 2002). This sequence may thus provide a mechanism for prostate carcinogenesis (Penta *et al*, 2001).

In this study, prostate tissue sections, analysed by light microscopy, showed infiltration of neutrophils associated with acute prostatic inflammation at 5 days after bacterial inoculation and an infiltration of lymphocytes and/or macrophages associated with chronic prostatic inflammation at 12 and 26 weeks after infection. Furthermore, prostate tissue sections showed an increase in the nuclear staining index of oxidative stress-associated marker, 8-OH-dG, and a decrease in *GSTP1* expression at 12 and 26 weeks after infection when compared with PBS-control prostate. These results support the hypothesis of De Marzo and his colleagues, and suggest that loss of *GSTP1* gene expression, probably as a result of hypermethylation of the CpG island sequences of *GSTP1*, will render the prostatic epithelial cells more vulnerable to genomic damage mediated by the infection itself or by the oxidant carcinogens elaborated by inflammatory cells (neutrophils, lymphocytes, and macrophages) (Nelson *et al*, 2003). Moreover, as suggested by De Marzo *et al* (1999b), the genomic DNA damage generated during chronic inflammation induced by bacterial insult may enhance the likelihood of neoplastic transformation.

In this inflammation-oxidative stress model, the progression of prostate epithelial cells from normal to neoplastic is marked by defined histological changes (De Marzo *et al*, 1999b; Nelson *et al*, 2002). The earliest lesion observed is PIA (De Marzo *et al*, 1999a, 1999b; Nelson *et al*, 2001), characterised by discrete foci of proliferative glandular epithelium with the morphological appearance of focal simple atrophy (Ruska *et al*, 1998) or postatrophic hyperplasia (Cheville and Bostwick, 1995), occurring in close association with chronic inflammation. An important feature of PIA is that the majority of lesions are associated with long-standing inflammation in the prostate, and many of the proliferating cells have an immature basal secretory cell phenotype similar to those found in PIN and CaP. Thus, this model strongly suggests that PIA associated with chronic inflammation may give rise to prostate carcinoma directly or indirectly by progression into high-grade PIN lesions (De Marzo *et al*, 1999a, 1999b, 2003; Nelson *et al*, 2001). In our study, chronic prostatic inflammation induced by *E. coli* bacteria was prominent at 12 weeks after infection, whereas acute inflammation subsided. Moreover, the chronic inflammation was more pronounced at 26 weeks after inoculation and was associated with severe dysplasia, characterised by complex glandular architecture and severe cytological atypia mimicking PIN or cancer-like lesions. Immunohistochemical analysis showed that the infected prostate had increased BrdU nuclear staining and a decrease in *p27*^*Kip1*^ expression compared with PBS-control prostate. These results support Virchow's hypothesis that chronic inflammation of long-standing duration can lead to cellular proliferation and cancer development (Balkwill and Mantovani, 2001). Moreover, the results suggest that proliferation of prostatic epithelial cells in the milieu of oxy-radical barrage may result in conversion of DNA damage to persistent mutations, some of which may provide prostatic cells with growth and/or antiapoptotic advantage. Therefore, chronic inflammation may be an initiator and a promoter of prostatic carcinogenesis through genotoxic and cytotoxic effects (Platz and De Marzo, 2004).

Overall, these results suggest that chronic prostatic inflammation may be a major factor in predisposition to prostatic carcinogenesis. During the malignant transformation of the prostate, AR may dissociate from heat-shock proteins and translocate into the nucleus, dimerise, bind to androgen-response elements in DNA, and activate several transcription genes involved in cell growth and survival (Debes and Tindall, 2004). It is well known that androgenic hormones and AR both have critical roles in normal prostate development function and in most prostate diseases, including CaP. Several clinical studies have shown that withdrawal of androgens leads to a rapid decline in CaP growth with significant clinical response. This response is short lived and tumour cells may re-emerge independently of androgen stimulation by acting through several mechanisms, including amplification of the AR gene or bypassing the AR by deregulating apoptotic genes. Amplification of the AR gene, accompanied by overexpression of the receptor, could lead to androgen-independent CaP-cell growth by increasing the sensitivity of CaP cells to low concentrations of circulating androgens (Debes and Tindall, 2004). Furthermore, by bypassing AR, both PTEN and the antiapoptotic gene bcl-2 have important roles in androgen-refractory CaP. Loss of PTEN activity reverses the inhibition of the phosphatidylinositol-3-kinase (PI3-K)-Akt pathway, permitting Bad phosphorylation by activated Akt. This PI3-K-Akt activation results in the release of Bcl-2, leading to cell growth and survival (Debes and Tindall, 2004). These two mechanisms are consistent with our observation of decreased expression of AR, loss of PTEN, and increased expression of BrdU incorporated into the newly synthesised DNA of replicating prostatic epithelial cells observed in the atypical prostatic gland mimicking PIN or cancer-like lesions as compared with normal and simple hyperplastic glands. Further molecular studies are needed to determine the mechanisms by which high-grade PIN induced by chronic bacterial infection and inflammation progresses towards an invasive carcinoma independently of circulating androgens and AR.

In the last decade, an increasing number of epidemiological and experimental studies have seemed to re-ignite the concept that long-standing chronic inflammation potentiates or promotes tumour development, growth, and progression. Long-standing chronic inflammation seems to have an active role in carcinogenesis and is now recognised as an important risk factor in the development of a number of malignancies with an infectious aetiology, such as stomach (*Helicobacter pylori*), liver (hepatitis B and C viruses), and colon cancer in patients with inflammatory bowel diseases (Ames *et al*, 1995; Giovannucci, 2001). It is also hypothesised that chronic inflammation may lead to the development and progression of prostatic carcinoma, although a direct causal role for chronic inflammation or infection in prostatic carcinogenesis is yet to be confirmed in humans.

Our study demonstrates that prostatic infection and inflammation results in focal prostatic glandular atypia (e.g., PIN and severe dysplasia), a potential precursor of prostatic adenocarcinoma. In addition, our results suggest a potential relationship between chronic inflammation and oxidative DNA damage that may have a role in prostate carcinogenesis. Further studies are needed to define the morphological and immunophenotypical changes within the prostate induced by chronic infection and to clarify a potential linkage between *E. coli* infection and inflammation in the aetiology of CaP. Moreover, *E. coli*-induced prostatitis will provide a valuable model system to determine the influence of host genetic factors on the development of CaP in an inflammatory milieu and to design new strategies for CaP prevention.

## Figures and Tables

**Table 1:** Prostate infection intensities in C3H/HeOuJ mice inoculated with PBS or *E. coli* bacteria after 5 days, 12 and 26 weeks

	**C3H/HeOuJ mice^a^**								
	**5 days**			**12 weeks**			**26 weeks**		
	***n* b**	**CFU^c^**	***P*-value^d^**	***n***	**CFU**	***P*-value**	***n***	**CFU**	***P*-value**
PBS	6	0	—	6	0	—	6	0	—
2 × 10^6^ *E. coli* bacteria	6	138	0.001	6	9375	0.001	6	30 785	0.001

Abbreviations: CFU=colony-forming unit; *E. coli*=*Escherichia coli*; PBS=phosphate-buffered saline.

a Mice infected with PBS or with *E. coli* bacteria on day 0.

b Number of mice infected in each experimental group.

c Geometric mean of CFU per mg of tissue for each experimental group at 5 days, and at 12 and 26 weeks after inoculation.

d *P*-values for comparisons of prostate CFU values between groups of *E. coli* infected and PBS mice were determined by Fisher's protected least significant difference test.
